# Sol-Gel Synthesis of New TiO_2_ Ball/Activated Carbon Photocatalyst and Its Application for Degradation of Three Hormones: 17α-EthinylEstradiol, Estrone, and β-Estradiol

**DOI:** 10.3390/toxics11040299

**Published:** 2023-03-24

**Authors:** El Mountassir El Mouchtari, Lekbira El Mersly, Kaltoum Belkodia, Anne Piram, Stéphanie Lebarillier, Samir Briche, Salah Rafqah, Pascal Wong-Wah-Chung

**Affiliations:** 1 Laboratoire Chimie Analytique et Moléculaire (LCAM), Faculté Polydisciplinaire de Safi, Université Cadi Ayyad, Marrakech 40000, Morocco; elmountassirelmouchtari@gmail.com (E.M.E.M.);; 2 Laboratoire Chimie Environnement (LCE), Centre National de la Recherche Scientifique (CNRS), Aix-Marseille University, 13000 Marseille, France; 3 Département Stockage de l’Energie et Revêtements Multifonctionnels (SERM), Moroccan Foundation for Advanced Science Innovation and Research (MAScIR), Rabat 10100, Morocco

**Keywords:** sol-gel, TiO_2_, activated carbon, photocatalysis, adsorption, hormones

## Abstract

Many approaches have been investigated to eliminate pharmaceuticals in wastewater treatment plants during the last decades. However, a lack of sustainable and efficient solutions exists for the removal of hormones by advanced oxidation processes. The aim of this study was to synthesize and test new photoactive bio composites for the elimination of these molecules in wastewater effluents. The new materials were obtained from the activated carbon (AC) of *Arganian spinosa* tree nutshells and titanium tetrachloride by the sol gel method. SEM analysis allowed one to confirm the formation of TiO_2_ particles homogeneously dispersed at the surface of AC with a controlled titanium dioxide mass ratio, a specific TiO_2_ anatase structure, and a highly specific surface area, evidenced by ATG, XRD, and BET analysis, respectively. The obtained composites were revealed to quantitatively absorb carbamazepine (CBZ), which is used as a referred pharmaceutical, and leading to its total elimination after 40 min under irradiation with the most effective material. TiO_2_ high content disfavors CBZ adsorption but improves its degradation. In the presence of the composite, three hormones (17α-ethinylestradiol, estrone, and β-estradiol) are partially adsorbed onto the composite and totally degraded after 60 min under UV light exposure. This study constitutes a promising solution for the efficient treatment of wastewater contaminated by hormones.

## 1. Introduction

Emerging contaminants (ECs) refer to a wide range of chemical and biological pollutants that have been recently detected in the environment and have the potential to cause adverse effects on human health and the environment [1,2,3]. These contaminants can arise from many sources such as industrial and agricultural activities, pharmaceuticals, personal care products, and household chemicals [4]. Their impacts on the environment can include effects on aquatic and terrestrial ecosystems, such as changes in species composition, reproductive and developmental abnormalities, and biodiversity declines. In addition, ECs can persist in the environment for long periods of time, leading to bioaccumulation and biomagnification in the food chain [5]. Estrone (E1), 17β-Estradiol (E2), and 17α-ethinylestradiol (EE2) are all forms of estrogen, which is a hormone primarily responsible for the development and regulation of the female reproductive system. However, estrogens also have other physiological roles in both males and females, including bone health, cognitive function, and cardiovascular health [6,7,8,9].

The concentrations of E1, E2, and EE2 in the environment can fluctuate depending on the location, source, and analysis method. However, studies have found that these estrogens can be present in the environment at low levels, measured in nanograms per liter (ng/L). For example, in the United States, it was shown that the concentrations of E1 and E2 in river water samples ranged from less than 1 to 65 ng·L^−1^, with an average concentration of 8 ng·L^−1^ [4]. EE2 was detected at concentrations ranging from less than 1 to 58 ng L^−1^, with an average concentration of 3 ng L^−1^. In Europe, the concentrations of E1, E2, and EE2 in surface water samples are of the same order of magnitude and comprise less than 1 to 85 ng·L^−1^, 1 to 49 ng·L^−1^, and 0.2 to 25 ng L^−1^, respectively [10,11,12]. It is important to note that even at very low concentrations, estrogens in the environment can have negative effects on aquatic wildlife, particularly fish and amphibians, which are sensitive to changes in hormone levels. Therefore, there is ongoing research and regulatory efforts aimed at reducing the discharge of estrogens into the environment and mitigating their effects [13,14,15].

The occurrence of organic pollutants in aquatic environments is mainly attributed to the discharge of wastewater effluents. Indeed, existing wastewater treatment plants (WWTPs) are not designed to efficiently and completely eliminate these organic pollutants [4,16]. Thus, several technologies have been developed to remove micropollutants from water and wastewater, e.g., adsorption by activated carbon [17,18,19], ozonation [20,21], and advanced oxidation processes (AOPs) have been developed to remove micropollutants from water and wastewater. AOPs are treatment technologies that involve the generation of highly reactive species, such as hydroxyl radicals, to degrade micropollutants. Among classical AOPs, UV/H_2_O_2_, Fenton’s reagent, and photocatalysis with TiO_2_ [22,23,24,25,26] can be cited, but other processes such as membrane filtration [27,28] and biological treatment [29,30] are also used. Overall, the micropollutant removal technology selection depends on several factors, including the type and concentration of micropollutants, the characteristics of the water or wastewater to be treated, and the treatment objectives. Most often, a combination of treatment technologies may be required to effectively remove micropollutants from water or wastewater. Even if some recent technologies allow for the complete removal of hormones, the formation of many by-products, sometimes more toxic than the initial one, remains questionable [31]. The use of activated carbon and TiO_2_ in a composite material (AC/TiO_2_) has shown promising results in removing micropollutants and their byproducts from both air and water due to the enhanced adsorption and photocatalytic capabilities of the composite material [32]. This technology is still under investigation and optimization and could be a valuable additional process to the suite of technologies available for micropollutant removal [33,34,35,36].

In line with the previous approach, the aim of this study is to propose a novel composite material made of TiO_2_ and bio-sourced activated carbon for the efficient photodegradation of some pharmaceuticals, specifically hormones in wastewater under UV light irradiation. Herein, the synthesis pathway of the materials is described, as well as their characterization by SEM, XRD, BET, and ATG analysis. In addition, kinetic studies were carried out to estimate the efficiency of AC/TiO_2_ composites for the removal of pharmaceutical products by both adsorption and photocatalysis and the role of TiO_2_ mass ratio.

## 2. Materials and Methods

### 2.1. Chemicals

Estrone (E1), 17β-Estradiol (E2), 17 α-Ethinyl-Estradiol (EE2), and carbamazepine (CBZ) with 99% purity were provided by Sigma-Aldrich as well as titanium tetrachloride, TiCl_4_, (99.99% purity), and hydrochloric acid (37%). Acetonitrile (HPLC grade), ethanol, and isopropanol (purity > 99.7%) were purchased from Fisher scientific SAS. Aqueous E1, E2, and EE2 solutions were prepared with a concentration equal to 10, 11.2, and 5.8 mg L^−1^, respectively, and carbamazepine at 50 mg L^−1^ with ultra-pure water from a Millipore device (Direct-Q^®^ 5 UV, Millipore SAS, Molsheim, France) after 24 h stirring at room temperature (25 °C).

### 2.2. Synthesis of AC/TiO_2_ Ball

Activated carbon was prepared as described in a previous work from *Arganian Spinosa* tree nutshells [36]. AC/TiO_2_ ball composite materials were synthesized by the sol-gel method. For this purpose, TiCl_4_ solutions (2.5, 1.29, and 0.85 M) were initially diluted in 10 mL of anhydrous isopropanol. After 5 min stirring at room temperature, 1 g of AC was added to each solution held under stirring. Then, 5 mL of distilled water was added dropwise. After 1 h of stirring at room temperature, the solvent was evaporated in a hood at room temperature and the solid was dried in the oven at 100 °C overnight.

The obtained solid materials were calcined at 500 °C in a muffle furnace for 2 h. Then, the powders were washed several times with boiling water to remove the remaining chloride ions and finally dried for 24 h at 100 °C.

### 2.3. Characterization of AC/TiO_2_ Ball

Scanning electron microscopy (SEM) using a FEI quanta 450 FEG operating at an accelerating voltage of 200 kV allowed us to observe the morphology of composite materials. TiO_2_ content in the composite was determined by thermogravimetric analysis (TGA) under air atmosphere using TGA Q5000 (TA 1000) equipment. The crystalline phase composition was determined by X-ray diffractometer using Bruker D8 Cu Kα radiation (k = 1.540593 nm) with a scan speed of 2° min^−1^. The surface area measurements of the materials synthesized were realized using a micromeritics 3 Flex apparatus.

### 2.4. Photocatalysis Experiment

The photocatalytic degradation of CBZ and hormones was carried out with an irradiation setup previously used by El Mouchtari et al. [35]. The adsorption capacity of the three AC/TiO_2_ ball composites was evaluated in a dark environment at 25 °C. Magnetic stirring was maintained until saturation of the material (adsorption–desorption equilibrium) for two hours. Samples were collected during the exposure at regular irradiation time. The residual concentrations were determined by high-performance liquid chromatography, and the degradation kinetics were plotted using the average data of triple experiments.

### 2.5. Adsorption Experiment

To determine the adsorption capacity of the composite material for CBZ, E1, E2, and EE2, we performed adsorption experiments at 25 °C. The solutions were left under magnetic stirring for 60 min to reach the adsorption–desorption equilibrium, and the equilibrium adsorption capacity *Q_e_* (mg g^−1^) was calculated using the following Equation 1:

(1) $$Q_{e}=\frac{\left(C_{0}-C_{e}\right)\times V}{W}$$

where *C_e_* (mg L^−1^) is the concentration of the drug product at equilibrium, *C*_0_ (mg L^−1^) is the initial concentration in the solution, *V* is the volume of the solution (L), and *W* is the mass of the composite material (g). The ayes it is dsorption kinetics were plotted using the average data of triple experiments.

### 2.6. Application in Wastewaters

Wastewater was collected from Aix-en-Provence WWTP (Pioline, France), which serves 175,000 inhabitants and operates through primary and biological treatment. The collected wastewater corresponds to the final effluent after the biological treatment. The wastewater was filtered through a 0.45 μm membrane filter using a Whatman glass microfiber filter, Binder-free GF/C 1.2 μm, and immediately stored in the dark at −20 °C. Before starting the treatment, we determined the organic and inorganic matter in the wastewater using total organic carbon (TOC) analysis with the liquid module (Analytik, Jena, German). We found that the water contains 21.8 and 34.7 mg L^−1^ of organic and inorganic carbon, respectively. For the irradiation experiments, the hormones were directly dissolved in wastewater effluent.

### 2.7. Experimental Analysis

The adsorption and degradation kinetics of hormones and CBZ were performed by HPLC (Perkin Elmer Altus 30). Chromatographic separation was obtained using a phenyl hexyl column (2.7 μm; 2.1 × 100 mm) distributed by the same supplier. A gradient method set at a flow rate of 0.5 mL min^−1^ and an oven temperature of 25 °C. The injected volume was 5 µL. Separation was achieved using a mixture of methanol/acetonitrile (65/35) (solvent A) and water (solvent B) with the following gradient: isocratic step at 90% B for 3 min, 90% B, 90% gradient at 0% B for 8 min, and isocratic step at 0% B for 3 min. UV detection at 285, 230, 210, and 280 nm was used to detect and quantify CBZ, E1, E2, and EE2, respectively. The accuracy of the method and the quality-control approach are presented in supporting information and in Table S1. Before injection, samples were systematically filtered on a 0.2 μm cellulosic filter of 15 mm in diameter, and the non-retention of pharmaceutical compounds on filters has been tested. The filter purchased by Agilent Technologies was used to remove the photocatalyst.

## 3. Results

### 3.1. Characterization of AC/TiO_2_ Ball

#### 3.1.1. Thermogravimetric Analysis

**Figure 1:**
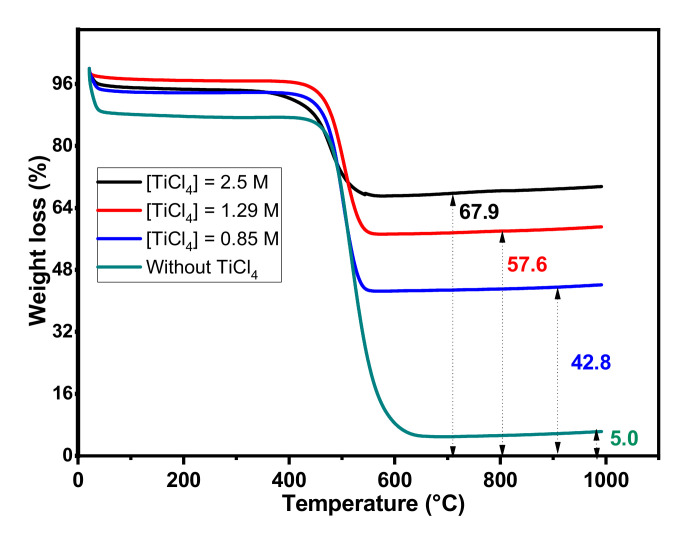
TGA profile of AC and AC/TiO_2_ ball composites with different TiCl_4_ concentrations.

Figure 1 shows the thermograms of AC and AC/TiO_2_ ball composites with different TiCl_4_ solutions. Systematically, whatever the material, two thermal events occur corresponding to two weight losses. The first one between 21 and 100 °C is probably due to the removal of water molecules adsorbed on the activated carbon surface, and the second one in the temperature range 100 to 1000 °C mainly corresponds to the combustion of carbonaceous materials.

The remaining weights indicated in Figure 1 suppose that almost all AC has disappeared, while the AC/TiO_2_ residual part increases with the concentration of TiCl_4_ solution. Consequently, it was assumed that the remaining weight in the composite corresponds to titanium dioxide formed during the sol-gel synthesis. Based on these results, the TiO_2_ contents of the synthesized composites were calculated as the difference between the remaining weight of AC/TiO_2_ ball and AC, and the values are gathered in Table 1.

The percentages of titanium dioxide determined by ATG (%TiO_2_ exp) show that the values obtained are very close to those expected (%TiO_2_ theo), highlighting the ability to control the TiO_2_/AC ratio by the sol-gel method as presented in Table 1.

#### 3.1.2. Crystallography

**Figure 2:**
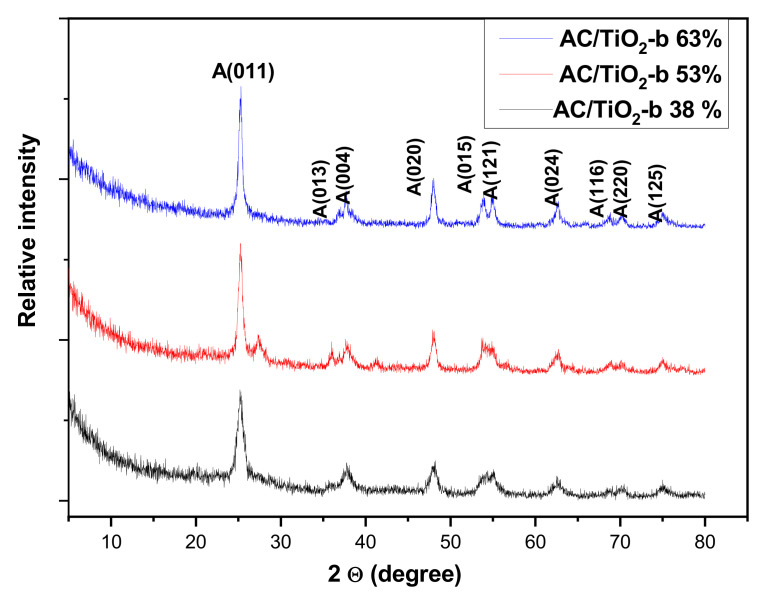
XRD patterns of the AC/TiO_2_ ball composites.

The X-ray diffractograms of the composite materials prepared from TiCl_4_ (TiO_2_ mass %: 38, 53, and 63) by sol-gel method are presented in Figure 2. It is observed that regardless of the amount of TiO_2_, the diffractograms exhibit similar diffraction patterns with characteristic peaks at angles of 25.18°, 36.9°, 37.86°, 48.17°, 53.83°, 55.03°, 62.64°, 68.91°, 70.25°, and 75.32°, corresponding to the reflections (011), (013), (004), (020), (015), (121), (024), (116), (220), and (125) of the anatase phase [37], respectively.

A weak peak at 27.3° corresponding to the (110) plane of the rutile phase [38] was observed only in the AC/TiO_2_-b 53% composite, which may have resulted from an unstable temperature during the calcination process at 500 °C. The classical Scherrer formula [36] was used to calculate the average crystallite size and percentage of anatase phase. The values are gathered in Table 1. From Table 1, it appears that the AC/TiO_2_ composites contain predominantly TiO_2_ with an anatase structure, the most photoactive one [39], and crystallites of a nanometric size around a few tenths of a nanometer on average, which help surface exchange.

#### 3.1.3. Morphology

**Figure 3:**
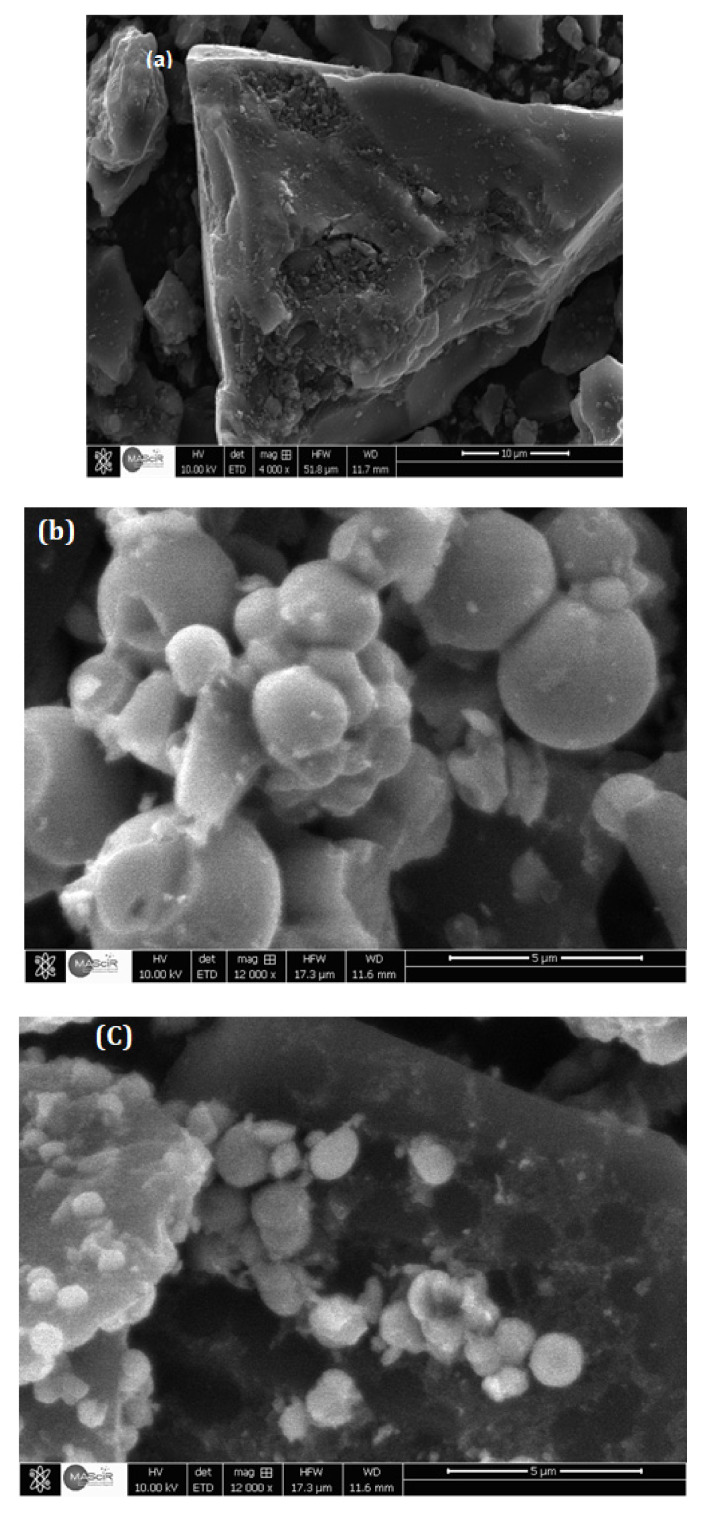

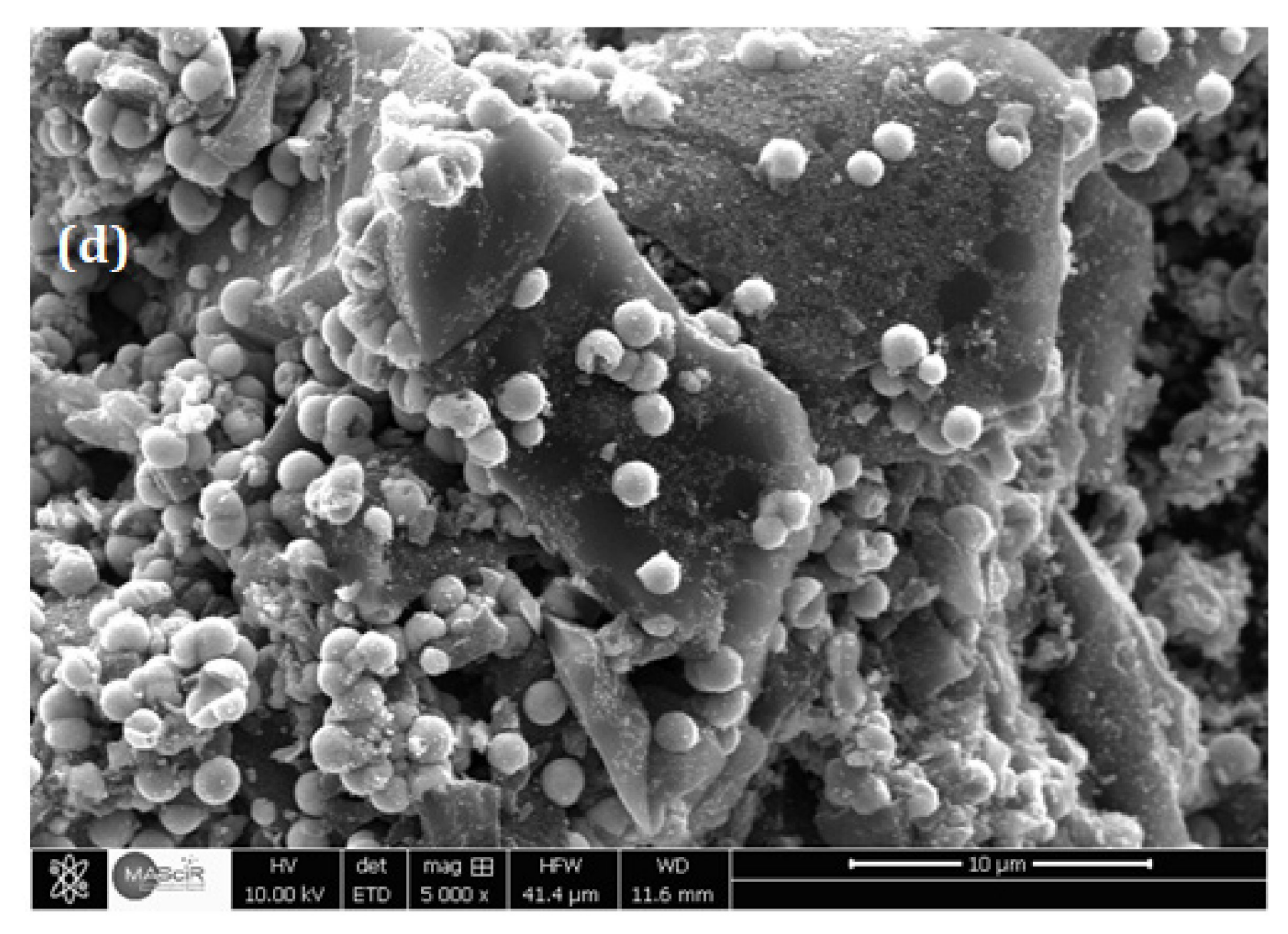
SEM image of AC and AC/TiO_2_ ball with different magnifications: (**a**) AC, (**b**) AC/TiO_2_-b 38%, (**c**) AC-TiO_2_-b 53%, and (**d**) AC/TiO_2_-b 63%.

Figure 3 shows the morphology of activated carbon and TiO_2_ composites obtained by SEM analysis with different magnifications. Figure 3a shows that the activated carbon particles have irregular shapes.

On the images of AC/TiO_2_-b 38% (Figure 3b), regular white beads (both solid and hollow) are observed on the surface of AC. One can also notice that grey nanobeads are deposited on the activated carbon surface for all three composites (Figure 3c).

As the TiO_2_ content increases, the size of the TiO_2_ particles grows and the agglomeration of white microbeads is favored, as seen in Figure 3b–d. In the same conditions, some AC surface areas contain TiO_2_ nanoballs homogeneously dispersed on the activated carbon surface (Figure 3d). These observations confirm XRD analysis and validate the production of titanium dioxide beads of both a micrometric (white bead) and nanometric (grey bead) size on the AC surface carbon for all three composites with the developed synthesis protocol.

**Figure 4:**
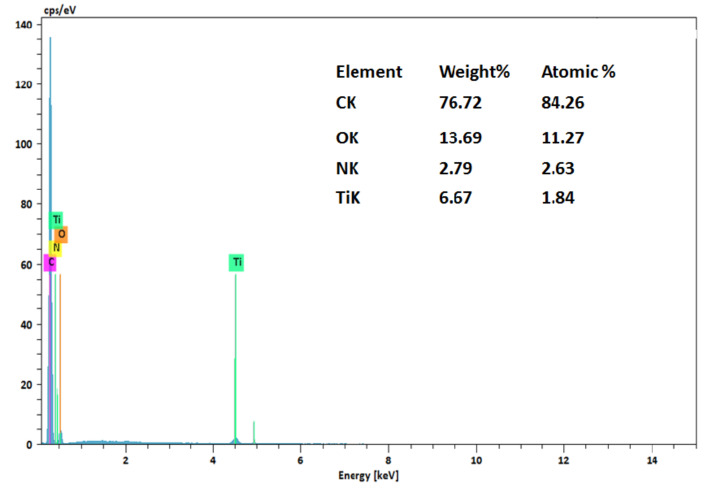
EDX spectrum of AC/TiO_2_-b 63%.

Elemental analysis of the AC/TiO_2_-b 63% composites was performed by energy dispersive X-ray spectroscopy (Figure 4). The local analysis qualitatively indicates the presence of TiO_2_ on the surface of AC and other elements, such as C, P, O, and N, which are constituents of the composition of AC.

#### 3.1.4. Surface Area and Pore Size

**Figure 5:**
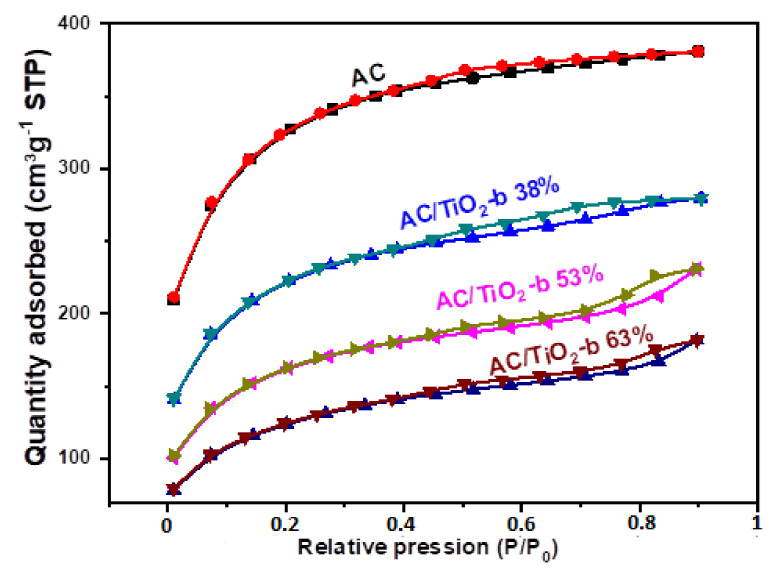
N_2_ adsorption/desorption isotherms of AC and AC/TiO_2_ composites.

The results from N_2_ adsorption–desorption isotherms are presented in Figure 4 and Table 2. Figure 5 shows that the isotherms fit with type I and IV isotherms, as described in IUPAC classification [40], which are typical for materials containing micro and mesopores. Moreover, the hysteresis of the photocatalysts is very uniform, suggesting that the pores have a conical shape [41,42,43].

Table 2 presents the textural properties of the activated carbon and composite materials. It can be observed that the addition of TiO_2_ leads to a significant decrease in the specific surface area and pore volume (maximum of 60%), while the pores diameter remains almost constant.

According to DRX analysis, most of the particles are of 52 to 77 nm size average (Table 1), and the maximum of the pores diameter distribution is located around 2.2 to 2.7 nm (Table 2). Consequently, it appears difficult to conceive that TiO_2_ particles can penetrate in AC pores, and this is justified by a weak change in pores diameter of the composite material compared to AC. Nevertheless, with the considered values being of an size average or maximum in diameter, an overlap of their size distribution may be possible, leading to the presence of some particles in AC large pores or their occlusion, justified by the porous volume decreases. This phenomenon is certainly negligible for small pores, and most TiO_2_ particles are immobilized on the surface of the activated carbon (and few of them in the pores). Therefore, it can be concluded that the addition of TiO_2_ to the composite did not significantly affect the pore nanostructure of the activated carbon.

### 3.2. Adsorption Equilibrium and Photodegradation Kinetic Studies

**Figure 6:**
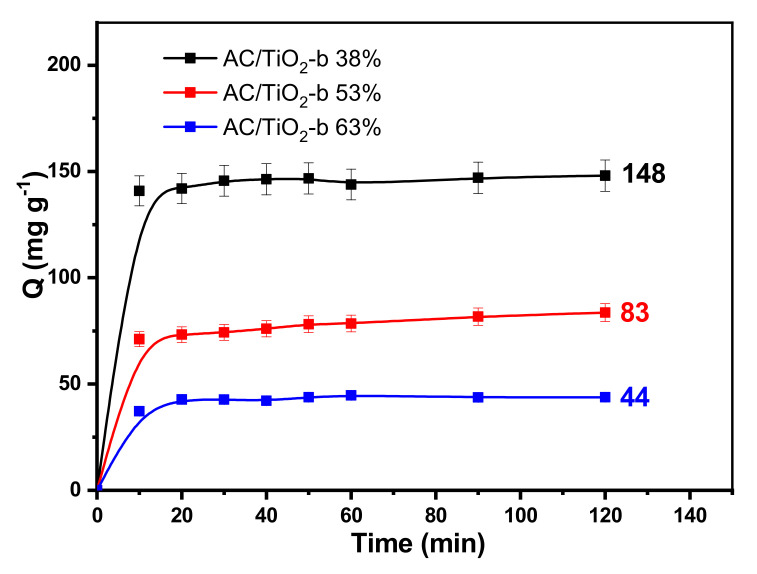
Concentration of CBZ adsorbed on AC/TiO_2_-b as a function of time, [CBZ] = 15 mg L^−1^, [AC/TiO_2_-b] = 0.1 g L^−1^, and T = 25 °C.

In a first approach, the efficiency of the composites has been carried out on CBZ used as a referred pharmaceutical. The effect of the contact time on CBZ adsorption reveals a two-step process as presented in Figure 6. CBZ adsorption takes place in the early stages of the experiment, and after approximately 60 min, a sorption equilibrium is reached whatever the composite. This typical adsorption profile is attributed to the large number of surface sites available at an initial time and the reduction in vacant sites on the surface for a longer contact time.

In addition, the results show that the amount of CBZ adsorbed is strongly affected by the TiO_2_ amount of the material. The composite with the lowest amount of TiO_2_ (AC/TiO_2_-b 38%) has the highest specific surface and total pore volume and therefore exhibits the highest adsorption capacity, with a maximum amount of CBZ adsorbed (Q) around 148 mg g^−1^. The data suggest a linear relationship between the adsorbed amount and the specific surface area as presented in Figure S1 in supporting information, in agreement with previous studies [34,36,44,45].

### 3.3. Photocatalytic Studies

**Figure 7:**
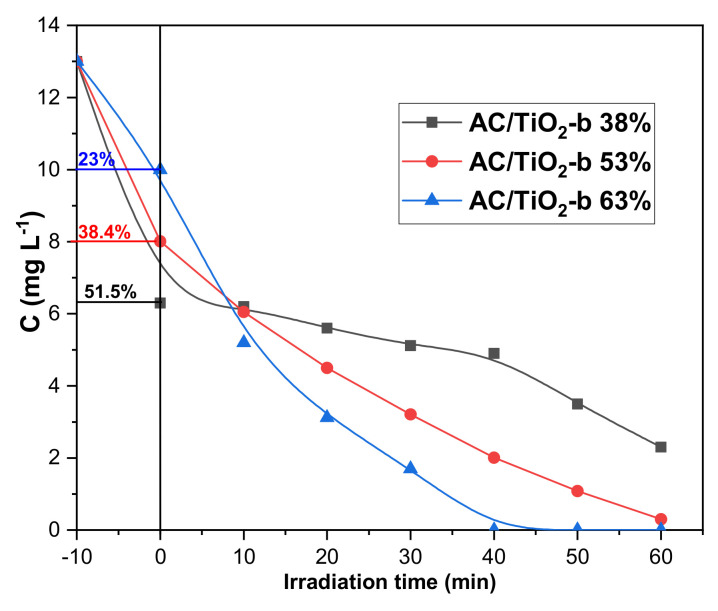
CBZ concentration in the presence of AC/TiO_2_ ball composites as a function of irradiation time: [CBZ] = 13 mg L^−1^, [AC/TiO_2_-b] = 0.1 g L^−1^ and Xe lamp.

The photocatalytic activity of the AC/TiO_2_ composites was undertaken using CBZ as the model organic substrate for pharmaceuticals and because of its well-known resistant to photochemical degradation [36]. After the adsorption step, in the presence of all composites, CBZ photodegradation appears to be very effective as shown in Figure 7. However, AC/TiO_2_-b 63% demonstrates a higher photocatalytic activity in comparison to the other composites since CBZ is completely removed within 40 min. The AC/TiO_2_-b 53% composite allows for CBZ total disappearance in 60 min, while on the same time scale a partial elimination of CBZ is observed with AC/TiO_2_-b 38%. The results suggest that TiO_2_ content promotes CBZ degradation.

### 3.4. Application on Wastewater Effluents

For this application, the AC/TiO_2_-b 53% composite was considered as the good compromise between all composite because of its demonstrated high adsorption and photodegradation on CBZ and its reasonable amount of TiO_2,_ making it costly to sustain.

**Figure 8:**
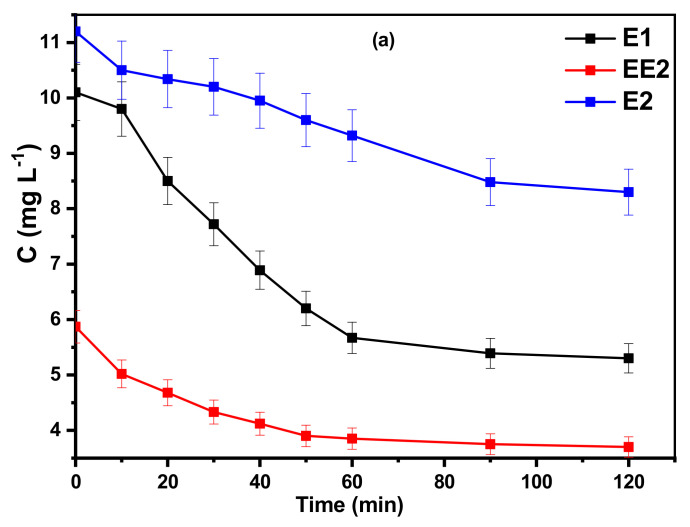

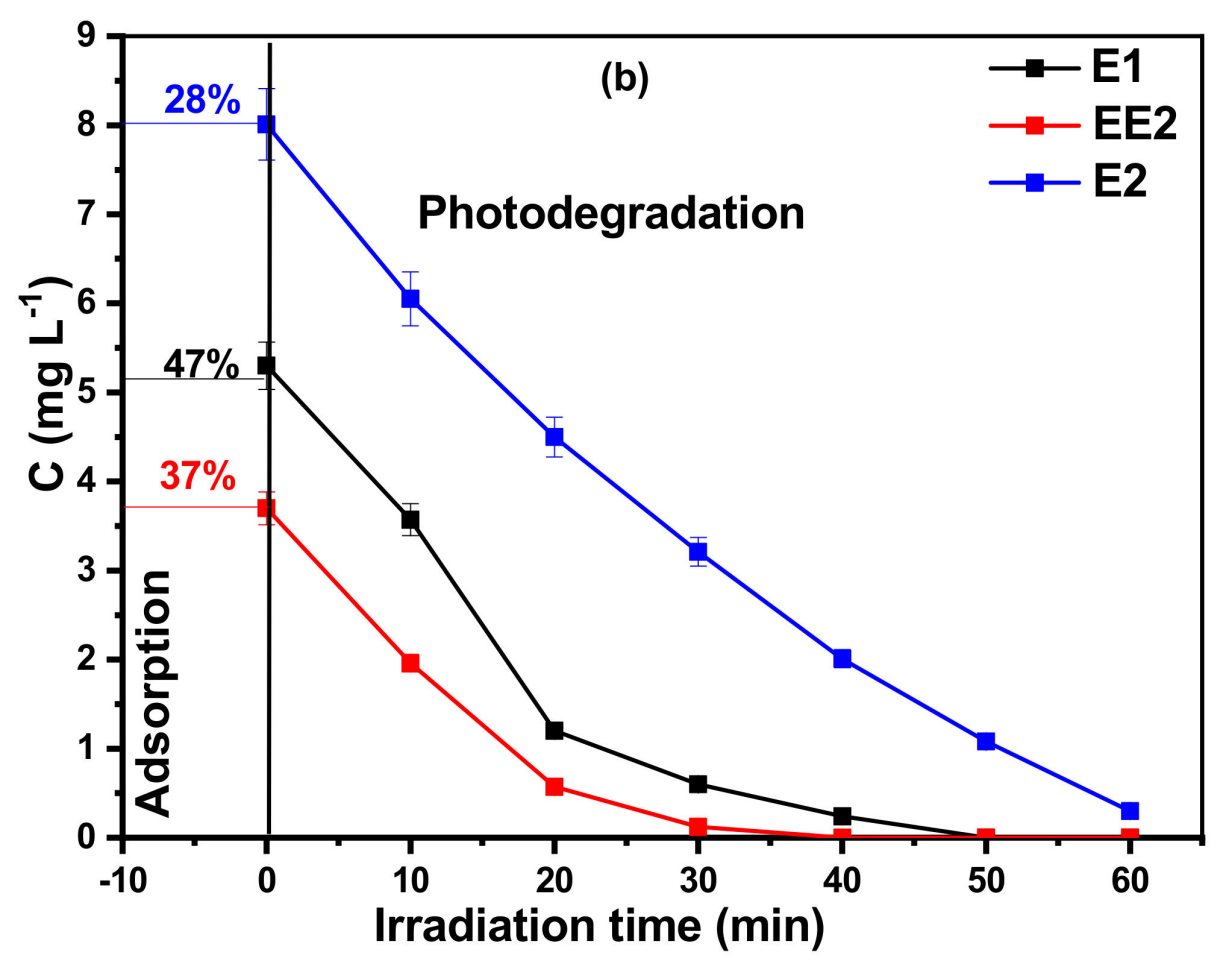
Hormone concentration as a function of time in the presence of AC/TiO_2_-b 53% and in wastewater effluent: (**a**) in the dark and (**b**) after the adsorption step and under UV light irradiation. [AC/TiO_2_-b 53%] = 0.13 g L^−1^, [E1] = 10 mg L^−1^, [EE2] = 5.8 mg L^−1^ et [E2] = 11.2 mg L^−1^, pH = 7, T = 25 °C, [O_2_] = medium, Xe lamp 300 W.

Assuming that the direct photodegradation of the hormones was negligible (Figure S2), their photodegradation in the presence of the composite was evaluated after stirring during 2 h in the dark to reach the adsorption/desorption equilibrium. The results of the adsorption study (Figure 8a) show that approximately, 28, 47, and 36% of E2, E1, and EE2, respectively, are removed from the effluents solution after 120 min. Thereafter, the photocatalytic degradation exhibits a high removal efficiency with a near complete disappearance of the hormones after only 60 min of irradiation time (Figure 8b).

The photocatalytic activity of AC/TiO_2_-b 53% was compared with the reported decontamination of hormones efficiency in ultrapure and wastewater using composite materials [46,47,48,49]. Thus, in pure and ultrapure water, the partial photocatalytic degradation of EE2 was observed depending on the exposure time and material: in the presence of the CaTiO_3_/WS_2_ heterostructure, 91% of degradation is reached after 120 min [46], and 90% in the presence of AgCl/ZnFe_2_O_4_ magnetic nanocomposite after the same irradiation time [48], while only 51% of EE2 elimination is measured in the presence of ZnFe_2_O_4_-Ag/RGO nanocomposite after 51 min exposure [45]. In wastewater, Yang et al. reported 50% of E1 transformation by a Fenton-like process after 90 min under irradiation using biosynthesized silver nanoparticles [47]. In our work, the new material composite significantly improves the photocatalytic degradation of E1 and EE2 hormones with their total removal in 60 min in wastewater effluent. However, it is important to note that there are limited data available on the photocatalytic degradation of a mixture of hormones, and further research is needed to evaluate the effectiveness of the AC/TiO_2_-b 53% composite in more complex wastewater systems. Nonetheless, these results suggest that photocatalytic degradation using the AC/TiO_2_-b 53% composite could be a promising and effective solution for the removal of pharmaceutical products from wastewater.

## 4. Conclusions

New composite materials based on bio-sourced activated carbon and TiO_2_ particles were successfully synthesized by the sol gel method using TiCl_4_ with controlled TiO_2_ structure, size, content, and phase. In addition, AC/TiO_2_ ball materials exhibit efficient adsorption and photocatalytic degradation of the model pharmaceutical compound and of carbamazepine and allow for the total removal of hormones such as estrogens. This study highlights the potential of using AC/TiO_2_ ball composites for the removal of pharmaceuticals and other emerging contaminants from wastewater, offering a promising solution for addressing water pollution and ensuring the protection of public health and the environment. Beyond the additional experiments to validate the efficiency of the process on other organic pollutants and determine optimized operational parameters, economic aspects should be studied in detail and compared to ongoing treatment in WWTP.

## Figures and Tables

**Table 1:** The theoretical and experimental mass composition (%TiO_2_ theo and %TiO_2_ exp), referred name, percentage of anatase and rutile structure percentage (anatase %, rutile %), and crystallite average size (TiO_2_ size) as a function of TiCl_4_ concentration.

[TiCl_4_] mol L^−1^	%TiO_2_ Theo	%TiO_2_ Exp	Referred Name	Anatase %	Rutile %	TiO_2_ Size (nm)
0.85	40.4	37.8	AC/TiO_2_-b 38%	100	0	52
1.29	50.7	52.6	AC/TiO_2_-b 53%	95	5	63
2.5	66.7	62.9	AC/TiO_2_-b 63%	100	0	77

**Table 2:** Textural properties of AC and AC/TiO_2_ composites, specific surface area (S_BET_), porous volume, and pore diameter.

Material	S_BET_ (m^2^ g^−1^)	Porous Volume (cm^3^ g^−1^)	Pores Diameter (nm)
AC	1070	0.59	2.2
AC/TiO_2_-b 38%	736	0.43	2.4
AC/TiO_2_-b 53%	541	0.35	2.6
AC/TiO_2_-b 63%	422	0.28	2.7

## Data Availability

Not applicable.

## Supplementary Materials

The following supporting information can be downloaded at https://www.mdpi.com/article/10.3390/toxics11040299/s1, Table S1: Relative standard deviation (RSD, n = 4 for CBZ et n = 6 for all the hormones), limit of quantification (LOQ) and detection (LOQ) for the four pharmaceuticals by HPLC analysis, Figure S1: CBZ adsorbed concentration at sorption equilibrium as a function of specific surface area of the composite, Figure S2: Concentration of hormones as a function of irradiation time, [E1] = 10 mg L−1, [EE2] = 5.8 mg L−1 and [E2] = 11.2 mg L^−1^, pH = 7, T = 25 °C, [O_2_] = Medium, lamp Xe 300 W, wastewater effluent.
