# Supplementary material for: Sol-Gel Synthesis of New TiO2 Ball/Activated Carbon Photocatalyst and Its Application for Degradation of Three Hormones: 17α-EthinylEstradiol, Estrone, and β-Estradiol
Source: Toxics. 2023 Mar 24;11(4):299. doi: 10.3390/toxics11040299 (PMC10143179; doi:10.3390/toxics11040299)
Supplement: Supplementary file 1 [file toxics-11-00299-s001.zip › toxics-2283477-supplementary.pdf]

Supplementary Materials

Quality Control and Accuracy

Two quality control criteria were used to guarantee the correct identification of the target compounds by HPLC: retention times matched those of the standard compounds within ± 0.1 minutes and signal-to-noise ratio was greater than three.

In addition, blank and standard were injected respectively every 2 and 10 sample analyses. All samples were injected at least three times. Two quality control criteria were used to guarantee the correct identification of the target compounds by LC: retention times matched those of the standard compounds within ± 0.1 minutes, selective response on the four UV detector channels (as presented in Tables 2 and 3). Any retention time shift in the chromatogram was corrected using the data on the standard analysis.

The accuracy of the method can be estimated through the relative standard deviation (RSD), the limit of quantification (LOQ) and detection (LOQ) values gathered in the following table.

Table S1. Relative standard deviation (RSD, n = 4 for CBZ et n = 6 for all the hormones), limit of quantification (LOQ) and detection (LOQ) for the four pharmaceuticals by HPLC analysis

Compound	RSD (%)	LOQ (mg L^{-1})	LOQ (mg L^{-1})
CBZ	4.8	0.044	0.013
E1	6.0	0.095	0.029
E2	1.6	0.042	0.013
EE2	9.3	0.143	0.043

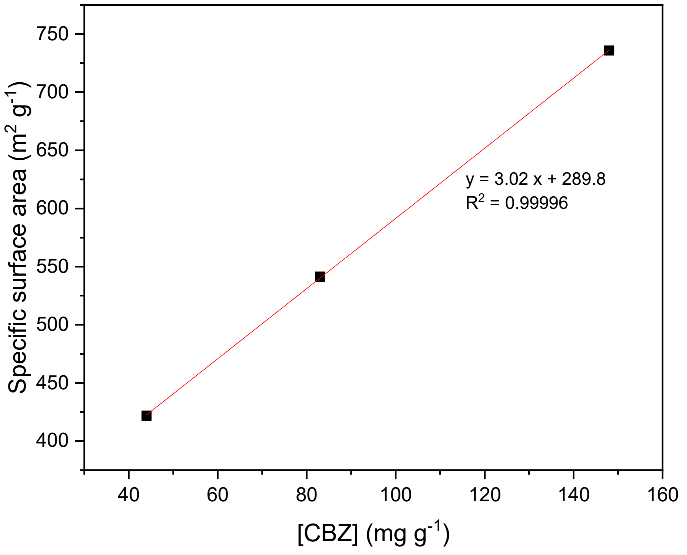

Figure S1. CBZ adsorbed concentration at sorption equilibrium as a function of specific surface area of the composite.

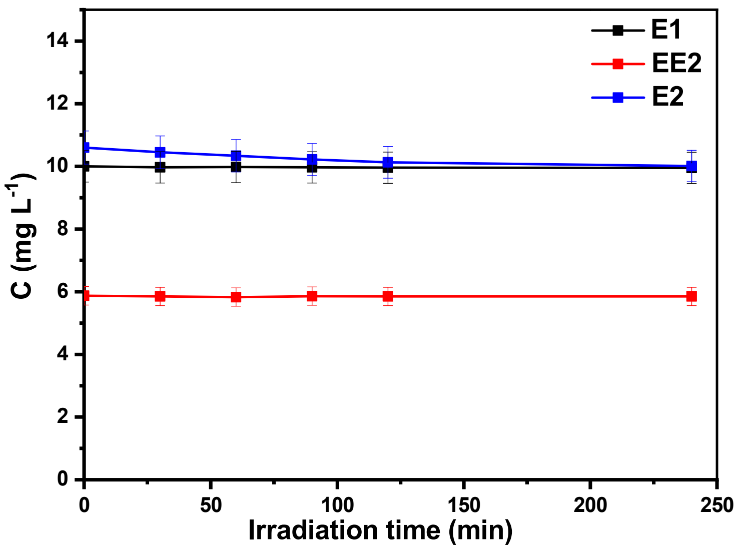

Figure S2. Concentration of hormones in wastewater effluent as a function of irradiation time, [E1] = 10 mg L^{-1}, [EE2] = 5.8 mg L^{-1} and [E2] = 11.2 mg L^{-1}, pH = 7, T = 25 °C, [O_2] = Medium, lamp Xe 300W.
